# Replicative senescence is associated with nuclear reorganization and with DNA methylation at specific transcription factor binding sites

**DOI:** 10.1186/s13148-015-0057-5

**Published:** 2015-03-04

**Authors:** Sonja Hänzelmann, Fabian Beier, Eduardo G Gusmao, Carmen M Koch, Sebastian Hummel, Iryna Charapitsa, Sylvia Joussen, Vladimir Benes, Tim H Brümmendorf, George Reid, Ivan G Costa, Wolfgang Wagner

**Affiliations:** Interdisciplinary Centre for Clinical Research (IZKF), RWTH University Medical School, Aachen, Germany; Institute for Biomedical Technology - Cell Biology, RWTH University Medical School, Aachen, Germany; Department of Hematology, Oncology, Hemostaseology and Stem Cell Transplantation, RWTH Aachen University Medical School, Aachen, Germany; Helmholtz-Institute for Biomedical Engineering, Stem Cell Biology and Cellular Engineering, RWTH University Medical School, Aachen, Germany; Institute for Molecular Biology, Mainz, Germany; Genomics Core Facility, European Molecular Biology Laboratory (EMBL), Heidelberg, Germany

**Keywords:** Senescence, Long-term culture, Telomeres, Epigenetic, DNA methylation, Transcription factor binding sites, Lamina, Massively parallel sequencing

## Abstract

**Background:**

Primary cells enter replicative senescence after a limited number of cell divisions. This process needs to be considered in cell culture experiments, and it is particularly important for regenerative medicine. Replicative senescence is associated with reproducible changes in DNA methylation (DNAm) at specific sites in the genome. The mechanism that drives senescence-associated DNAm changes remains unknown - it may involve stochastic DNAm drift due to imperfect maintenance of epigenetic marks or it is directly regulated at specific sites in the genome.

**Results:**

In this study, we analyzed the reorganization of nuclear architecture and DNAm changes during long-term culture of human fibroblasts and mesenchymal stromal cells (MSCs). We demonstrate that telomeres shorten and shift towards the nuclear center at later passages. In addition, DNAm profiles, either analyzed by MethylCap-seq or by 450k IlluminaBeadChip technology, revealed consistent senescence-associated hypermethylation in regions associated with H3K27me3, H3K4me3, and H3K4me1 histone marks, whereas hypomethylation was associated with chromatin containing H3K9me3 and lamina-associated domains (LADs). DNA hypermethylation was significantly enriched in the vicinity of genes that are either up- or downregulated at later passages. Furthermore, specific transcription factor binding motifs (e.g. EGR1, TFAP2A, and ETS1) were significantly enriched in differentially methylated regions and in the promoters of differentially expressed genes.

**Conclusions:**

Senescence-associated DNA hypermethylation occurs at specific sites in the genome and reflects functional changes in the course of replicative senescence. These results indicate that tightly regulated epigenetic modifications during long-term culture contribute to changes in nuclear organization and gene expression.

**Electronic supplementary material:**

The online version of this article (doi:10.1186/s13148-015-0057-5) contains supplementary material, which is available to authorized users.

## Background

Primary cells lose proliferative potential during *in vitro* culture and enter a senescent state after a limited number of cell divisions [1]. For example, fibroblasts and mesenchymal stromal cells (MSCs) undergo continuous morphologic and functional changes in the course of culture expansion. These include an increase in cell size and loss of *in vitro* differentiation potential [2,3]. Additionally, as MSCs are used in many clinical trials, standardization and quality control are prerequisites for the development of cellular therapeutics. It is therefore important to define the state of cellular aging in cell preparations and to better understand the mechanisms that elicit these dramatic changes during *in vitro* culture.

A reduction in telomere length has a definitive role in the loss of chromosomal integrity during culture expansion [4,5]. The nuclei of senescent cells reveal further structural changes, such as the development of senescence-associated heterochromatin foci (SAHF) [6], the formation of γH2AX-foci associated with DNA damage and double-strand breaks [7], and distorted organization of nuclear lamina [8]. Chromosomes are not randomly organized within the nucleus, but have a preferred position in relation to specific neighboring chromosomes [9,10]. Reorganization of chromosomal territories has been associated with changes in the epigenetic regulation of gene expression [11] and consequently may also be implicated in functional changes resulting from long-term culture of primary cells.

Recent evidence suggests that replicative senescence is accompanied by epigenetic modifications at specific CpG sites [12-14]. Senescence-associated DNA methylation (SA-DNAm) changes are very similar in both fibroblasts and MSCs [14,15] reflecting that both cell types may be closely related [16]. It has been suggested that long-term culture *in vitro* is associated with global DNA hypomethylation, whereas local DNA hypermethylation occurs at specific CpG sites [17]. SA-DNAm changes are related to, but not identical with, age-associated DNAm changes [12,15]. SA-DNAm changes, as well as age-associated DNAm changes are enriched in developmental genes, such as homeobox genes [12], coincide with polycomb group target genes [18,19] and with specific histone marks [13,20]. However, it is unclear how these changes in DNAm patterns are governed and if they are functionally relevant.

Two non-exclusive mechanisms may influence SA-DNAm changes: 1) compatible with the perception of epigenetic drift [21,22], they might result from loss of control at circumscribed genomic regions or 2) DNAm changes are directly controlled by regulated protein complexes (for example, DNA methyltransferases) targeting specific regions in the genome. In this study, we characterized nuclear changes during long-term culture of human fibroblasts and MSCs with particular focus on changes in nuclear morphology, telomere distribution, DNAm, and gene expression changes, to gain further insight in the underlying processes of senescence.

## Results

### Telomeres shift to the nuclear center during expansion in culture

**Figure 1:**
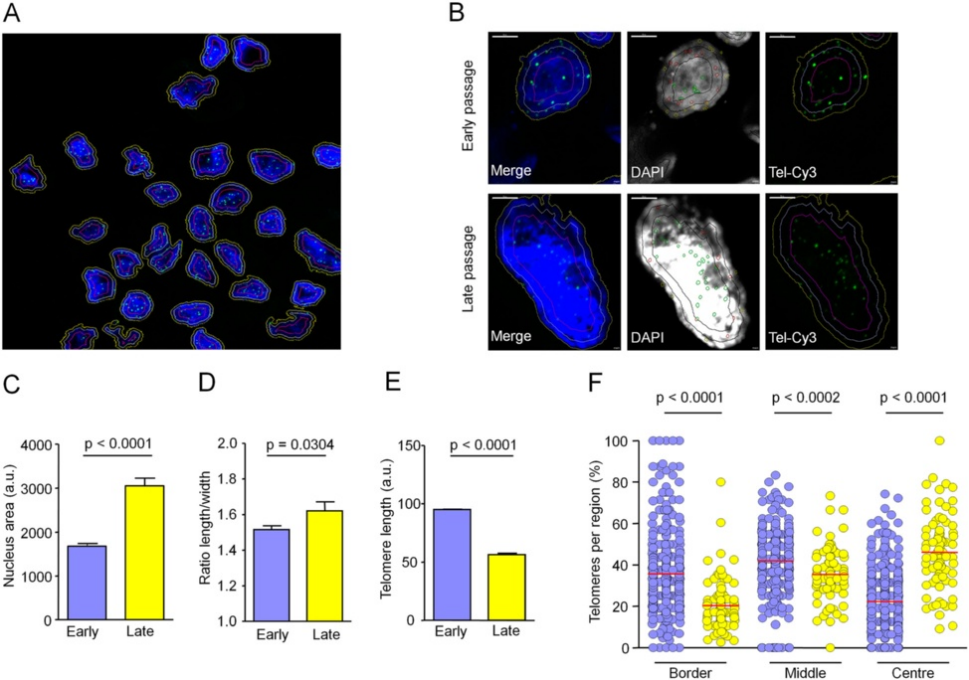
**Telomere distribution in senescent fibroblasts.** Telomeres were analyzed by Q-Fish (labeled with Cy3) in nuclei of fibroblasts of early or late passage (*n* = 3). The nuclear region was counterstained with DAPI. An overview of a cytospin **(A)** and enlarged nuclei at early and late passage **(B)** are exemplarily depicted. Separation of nuclear zones in the border, middle, and center is indicated by yellow, white, and violet lines, respectively (size bar = 5 μm). Overall, the nuclear area was greatly increased in cells of late passage **(C)** and nuclei became more elongated **(D)**. Telomere length markedly decreased in fibroblasts of late passage **(E)** (*a.u.* = arbitrary units; error bars depict standard error of nuclei analyzed; early passage: 374 nuclei; late passage: 151 nuclei). The distribution of telomeres changed upon senescence: in early passages (purple dots), they were primarily localized in border and middle regions, whereas distribution changed towards the nuclear center in late passages (yellow dots, data from three biological replica, *t*-test in all statistical analyses) **(F)**.

Nuclei and telomeres were analyzed in human fibroblasts at early (P3 to P5) and corresponding late passages (P21 to P40) with regard to nuclear area and by quantitative fluorescent *in situ* hybridization (Q-Fish) with telomere repeat probes (Figure 1A,B). Overall, nuclear area, as defined by optical sections, increased significantly during culture expansion (*P* < 0.0001; *t*-test; Figure 1C), whereas nuclear thickness remained relatively constant (5 to 7 μm in *z*-stacks). Furthermore, nuclei acquired an elongated morphology (Figure 1D). As anticipated, telomere length decreased at later passages (*P* < 0.0001; Figure 1E). Localization of telomeres within the nucleus was segmented into either the peripheral region, middle region, or central region [23]. In early passages, telomeres were predominantly localized at border regions close to the nuclear lamina while they appeared to be redistributed to the nuclear center at later passages (Figure 1F). Changes in nuclear size, morphology, and localization of telomeres reflect chromosomal reorganization during *in vitro* culture expansion.

### Analysis of senescence-associated DNAm

**Figure 2:**
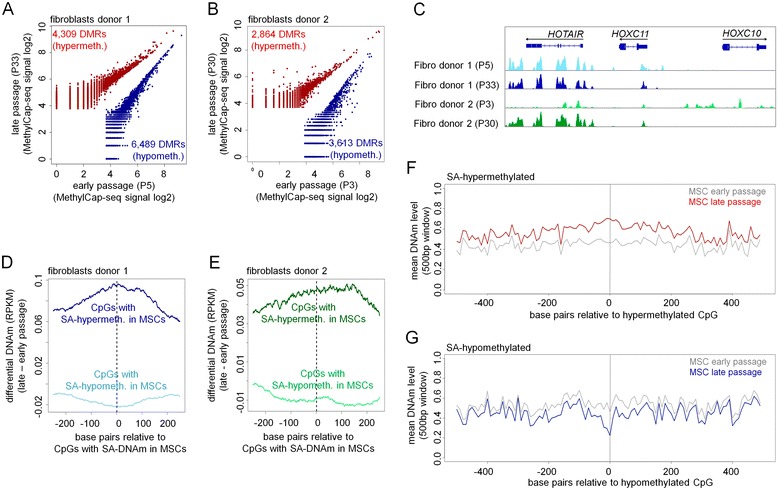
**Senescence-associated DNAm changes.** Scatter plots of global DNAm profiles (analyzed by methyl-capture sequencing) of early *versus* late passage are depicted for fibroblasts of donor 1 **(A)** and 2 **(B)** (*DMR* = differentially methylated region; log2 signal intensities are depicted for each DMR). Prominent DMRs were observed within the *HOXC* locus **(C)**. Senescence-associated DNAm changes in fibroblasts (MethylCap-seq data) were then compared to DNAm changes upon long-term culture of MSCs (450k IlluminaBeadChip) of our previous work [14]. Differential DNAm levels (late minus early passage) in fibroblast 1 **(D)** and 2 **(E)** were analyzed at genomic regions surrounding the CpGs with senescence-associated DNAm changes in MSCs (450k IlluminaBeadChip data is plotted). This comparison reflected the overlap of senescence-associated DNAm changes between the two methods and cell types. Subsequently, we analyzed the mean DNAm level of neighboring CpGs in the 450k IlluminaBeadChip data of MSCs. We used a 500-bp window in the vicinity of CpGs with significant SA-hypermethylation **(F)** and SA-hypomethylation **(G)**.

DNAm patterns were measured in two fibroblast preparations at early (P3 or P5) and late passages (P30 or P33). To this end, we used MethylCap-seq, which is based on capturing methylated DNA with the methyl-CpG-binding domain (MBD) of the methyl-CpG-binding protein 2 (MeCP2) and subsequent next-generation sequencing of salt-eluted DNA [24]. This analysis revealed that differentially methylated regions (DMRs) occur during culture expansion: 4,309 and 2,864 regions became hypermethylated and 6,489 and 3,613 regions hypomethylated during culture expansion of donor 1 and donor 2 cells, respectively (Figure 2A,B). Regions within the *HOXC* locus revealed prominent DMRs, particularly in donor 2 (Figure 2C). However, the overlap of DMRs between the two donors was only 3.99% and 4.24% for hyper- and hypomethylated regions, respectively. This was in contrast to overlapping methylation changes observed during long-term culture of fibroblasts and MSCs when using either IlluminaBeadChip Technology [12,14,19] or whole-genome single-nucleotide bisulfite sequencing [17].

Therefore, we compared the results from MethylCap-seq with our recent study on senescence-associated (SA-) DNAm changes in MSCs using 450k IlluminaBeadChips [19]. With IlluminaBeadChips, 1,702 CpGs were found to be significantly hypomethylated upon long-term culture and 2,116 CpGs became hypermethylated (adjusted *P* value < 0.05 and DNAm change > 20%). MethylCap-seq signals were then analyzed in a 600-bp window around these SA-DNAm changes identified by BeadChip technology. Overall, differential MethylCap-seq signals had the same tendency of SA-DNAm changes as observed with the 450k IlluminaBeadChip data (Figure 2D,E). This finding confirmed that senescence-associated DNAm changes identified by IlluminaBeadChip technology are also present in MethylCap-seq data of fibroblasts.

Subsequently, we analyzed whether SA-DNAm changes were restricted to individual CpGs or if adjacent CpGs were also affected. We focused on the most significant CpGs of the 450k IlluminaBeadChip data (1,702 and 2,116 CpGs) and found that SA-hypermethylation and hypomethylation was not only restricted to individual CpGs but also occurred in upstream and downstream CpGs, usually within a region of 500 bp (Figure 2F,G). There were fluctuations in mean DNAm level in the vicinity of CpGs with the most significant SA-DNAm changes, which cannot be resolved by analysis of DNA fragments in MethylCap-seq. Therefore, analysis of DNAm at single-nucleotide resolution using IlluminaBeadChip technology or genome-wide bisulfite sequencing might be advantageous for analysis of site-specific changes during culture expansion.

### Senescence-associated DNAm coincides with histone marks and lamina-associated domains

**Figure 3:**
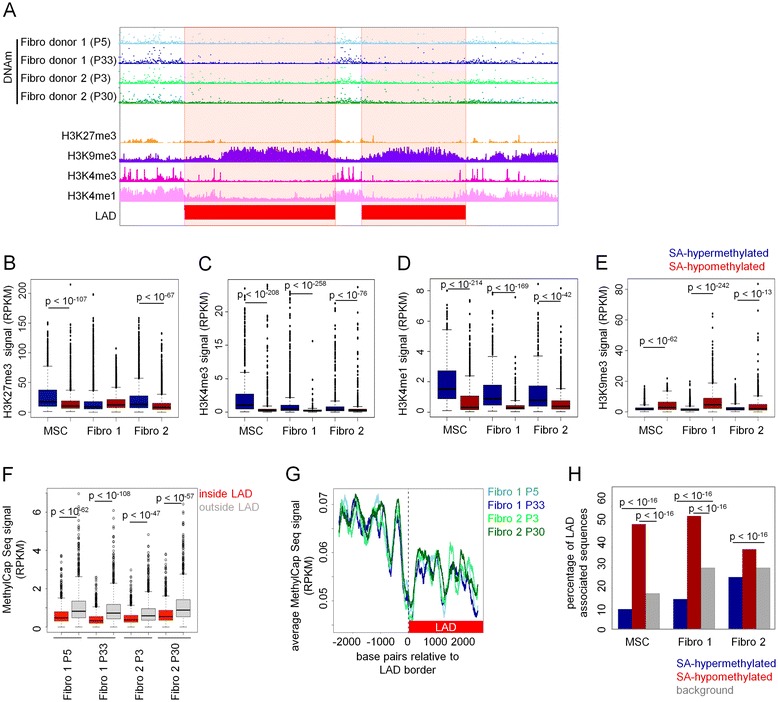
**Senescence-associated hypomethylation is enriched in lamina-associated domains.** DNAm profiles of fibroblasts at early and late passage (MethylCap-seq) were compared to previously published data on H3K27me3 [30], H3K4me3 [30], H3K4me1 [30], H3K9me3 [30], and lamina-associated domains (LADs) [29] in fibroblasts. Non-methylated DNA was particularly associated with the histone mark H3K9me3 and LADs, whereas H3K27me3, H3K4me3, and H3K4me1 were significantly reduced in these regions. RPKM signals are exemplarily depicted for a region in chromosome 16 **(A)**. Distributions of average RPKM levels of H3K27me3 **(B)**, H3K4me3 **(C)**, H3K4me1 **(D)**, and H3K9me3 **(E)** in 1,000-bp windows around DMRs are shown. Average signal intensity of DNAm was significantly lower inside LADs than outside LADs (Mann-Whitney test of equal means) **(F)**. A particular sharp decline of DNAm level was observed at the border of LADs (in all samples) **(G)**. Senescence-associated DMRs were then correlated with LADs. The proportion of senescence-associated (SA) hypermethylation was significantly decreased in LADs while SA-hypomethylation was highly significantly increased in LADs as compared to randomly selected regions (two-tailed Fisher’s Exact test) **(H)**.

We then compared DNAm changes resulting from long-term culture with previously published datasets on posttranslational histone modifications [25] and on lamina-associated domains (LADs) [26] in human fibroblasts (Figure 3A). Genomic regions with SA-hypermethylation in late-passage samples from fibroblasts and MSCs revealed significant enrichment in regions with trimethylation on histone 3 at lysine 27 (H3K27me3; not observed in fibro 1), trimethylation on histone 3 at lysine 4 (H3K4me3), and monomethylation on histone 3 at lysine 4 (H3K4me1) (Figure 3B,C,D). H3K27me3 is characteristic for inactivated chromatin within gene-rich regions, while H3K4me3 and H3K4me1 are, respectively, indicative of active promoters and enhancers [27]. In contrast, H3K9me3, a repressive histone mark mainly occurring in gene-poor regions, was associated with non-methylated regions. All samples analyzed had a significant presence of SA-hypomethylation in genomic regions with H3K9me3 marks (Figure 3E). Thus, specific histone modifications are enriched in regions with DNAm changes during long-term culture.

Subsequently, we compared our DNAm datasets with a high-resolution map of genomic interaction sites with the nuclear lamina in human fibroblasts, which comprises 1,239 genomic regions representing about 40% of the human genome [26]. DNAm levels were lower in lamina-associated regions than in the remaining genomic regions (Figure 3F), and similar findings were recently described [17]. Conversely, H3K27me3 marks were particularly observed outside of LADs, whereas H3K9me3 marks were more prevalent inside LADs as described before [26]. LAD borders clearly demarcate the level of DNAm (Figure 3G). We correlated senescence-associated DMRs with LADs and found that hypomethylated sites were enriched inside LADs while hypermethylated sites were enriched outside of LADs. Similar results were observed with SA-DNAm changes in MSCs, which were determined by 450k BeadChip technology (Figure 3H). We therefore postulated that a shift of lamina association, particularly at the border regions of LADs, may contribute to DNAm changes during culture expansion. However, the SA-DNAm changes were not related to the borders of LADs. In summary, loss of DNAm during culture expansion is especially observed in heterochromatin associated with the nuclear lamina, whereas DNA hypermethylation was observed in regions not associated with the lamina.

### Gene expression changes during replicative senescence

**Figure 4:**
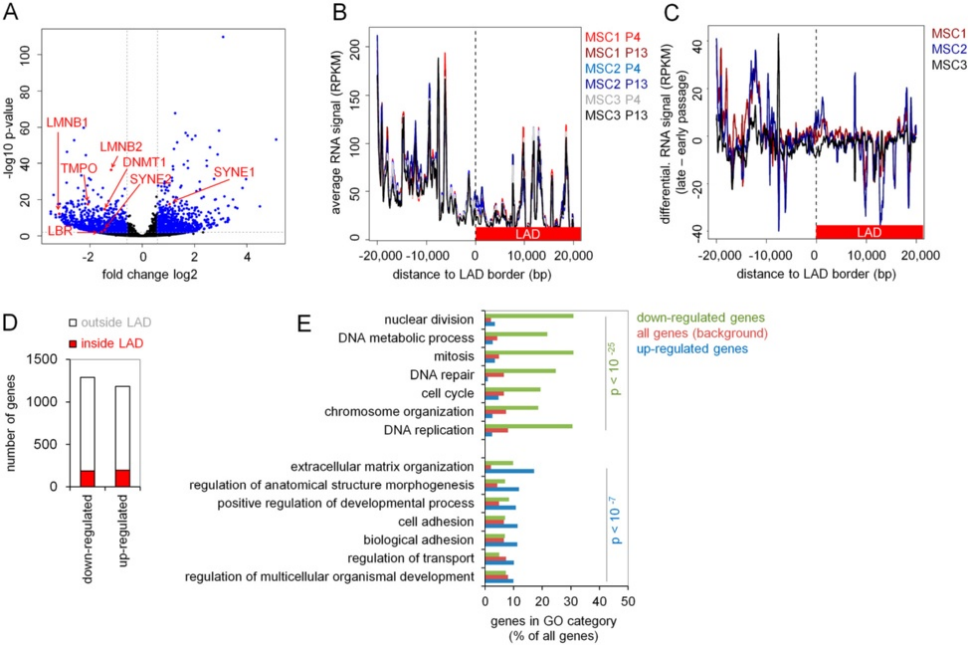
**Gene expression changes upon long-term culture.** Gene expression profiles (RNA-seq) were analyzed in MSCs of early passage (P4) and late passage (P13; *n* = 3). The volcano plot demonstrates differential expression upon long-term culture. Relevant genes are indicated in red **(A)**. Particularly, genes localized at the border of LADs were hardly expressed **(B)**. No significant association of senescence-associated gene expression changes was observed at the border of LADs **(C)**. Differentially expressed genes are in genomic regions that are not localized in LADs **(D)**. Gene ontology (GO) analysis was performed for genes, which were either significantly up- or downregulated compared to all genes (Fisher’s Exact test followed by Benjamin Hochberg multiple test correction). Shown are the most significant categories **(E)**.

To further correlate DNAm changes with gene expression changes, we sequenced the transcriptome of three MSC preparations at early (P3) and late passages (P13; the same MSC preparations previously used for the analysis of DNAm profiles [19]). Six hundred forty-eight genes were downregulated and 499 genes upregulated during long-term culture (FDR < 0.01 and log2 fold change > 2; Figure 4A; Additional file 1: Table S1). Interestingly, among the significantly downregulated genes were lamin B1 (*LMNB1*; *P* = 5.9*10 − 13) and lamin B2 (*LMNB2*; *P* = 4.1*10^−37^). Further, downregulated genes included the lamin B receptor (*LBR*; *P* = 6.8*10^−4^), which anchors the lamina and heterochromatin to the membrane; thymopoietin (*TMPO*; *P* = 3.8*10^−18^), which may play a role in the assembly of the nuclear lamina and thus help maintain the structural organization of the nuclear envelope; and spectrin repeat containing nuclear envelope 2 (*SYNE2*, *P* = 0.005), whereas *SYNE1* was upregulated (*P* = 3.1*10^−18^). These results indicate that differential expression of genes involved in the nuclear lamina may contribute to reorganization of chromatin during long-term culture.

Subsequently, we analyzed whether genes localized within the LADs are particularly affected by senescence. Overall, these genes were less expressed, especially at the border of LADs (Figure 4B), which is in agreement with previous findings [26]. However, gene expression changes during culture expansion were not related to LADs or to the border of LADs (Figure 4C). The number of upregulated and downregulated genes was similar between LADs and non-lamina-associated regions (Figure 4D). Thus, neither the hypomethylation in LADs nor gene expression changes during culture expansion seem to be triggered by extension or restriction of chromatin interaction sites with the nuclear lamina.

Subsequently, we performed gene ontology (GO) analysis of differentially expressed genes to gain better insight in the functional changes during long-term culture: downregulated genes revealed a highly significant enrichment in categories involved in cell division and DNA repair whereas upregulated genes were enriched in cell adhesion, development, and extracellular matrix organization (Figure 4E). This is in line with our previous reports using microarray analysis of RNA profiles in culture expansion [2]. We compared changes in the DNAm pattern upon culture expansion with differential gene expression. Overall, hypermethylated regions were enriched in the vicinity of up- and downregulated genes while hypomethylated regions were not enriched in the vicinity of up- or downregulated genes (Table 1). These findings suggest that hypermethylation of specific genomic regions impacts on gene expression changes during culture expansion.

**Table 1:** **Association of DMRs and gene expression changes using the projection test**

**DNAm**	**Gene expression**	**Enriched/depleted**	***P*** **value**
SA-hyperFibro1	Downregulated genes	Enriched	2.44E-08
SA-hyperFibro2	Downregulated genes	Enriched	1.88E-05
SA-hyperMSC	Downregulated genes	Enriched	1.30E-07
SA-hyperFibro1	Upregulated genes	Enriched	0.011
SA-hyperFibro2	Upregulated genes	Enriched	2.07E-09
SA-hyperMSC	Upregulated genes	Enriched	0.006
SA-hypoFibro1	Upregulated genes		No significance
SA-hypoFibro2	Upregulated genes		No significance
SA-hypoMSC	Upregulated genes		No significance
SA-hypoFibro1	Downregulated genes	Depleted	0.0008
SA-hypoFibro2	Downregulated genes		No significance
SA-hypoMSC	Downregulated genes		No significance

### Transcription factor binding sites in senescence-associated DMRs

**Figure 5:**
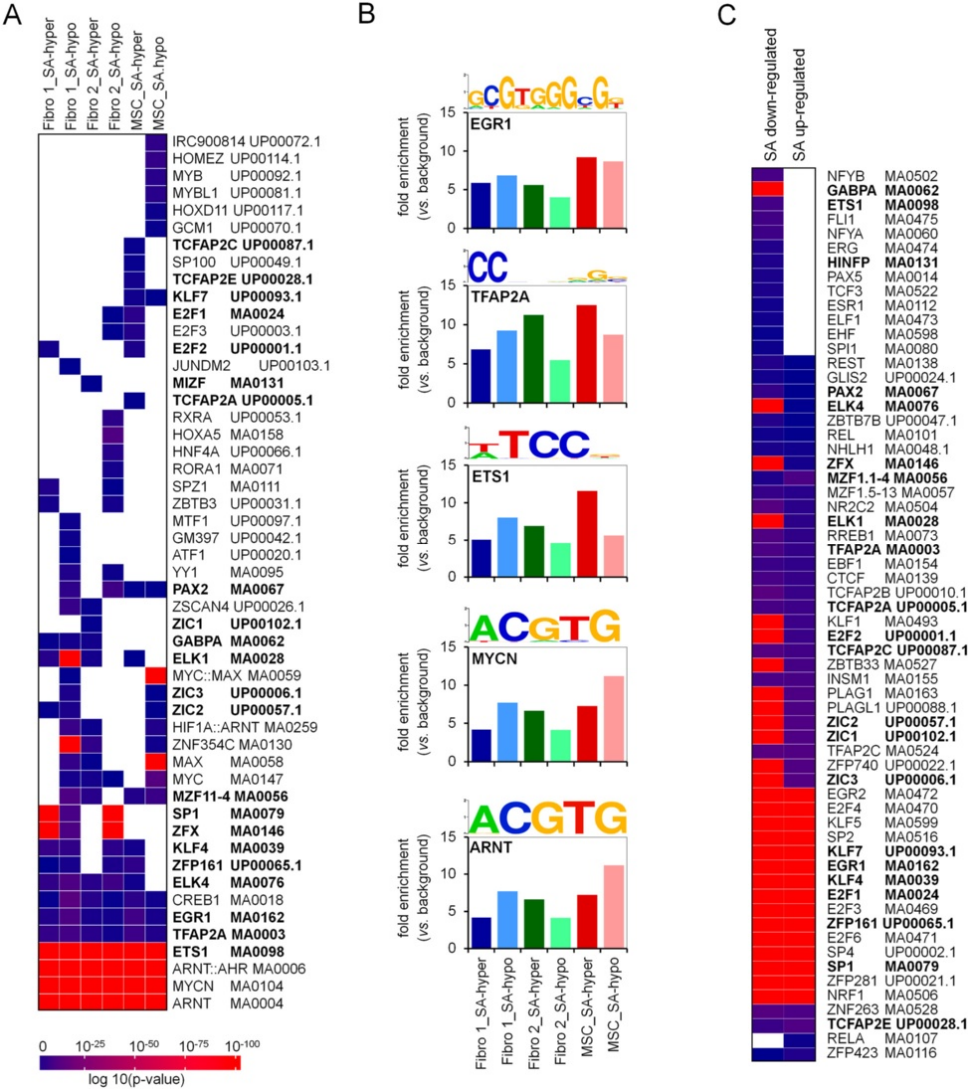
**Transcription factor binding sites in differentially methylated regions**. Analysis of transcription factor binding sites was performed in DMRs and promoter regions of up-/downregulated genes. The heatmap shows the -log10 *P* value for motifs enriched in at least one DMR signature (Fisher’s Exact test followed by Benjamin Hochberg multiple test correction, adj. *P* value < 0.05) **(A)**. Motifs for the five most significant transcription factors are depicted **(B)**. Subsequently, binding sites in promoter regions of either up- or downregulated genes were analyzed, and motifs common in both lists are marked in bold (Fisher’s Exact test followed by Benjamin Hochberg multiple test correction, adj. *P* value < 0.05) **(C)**.

Next, we performed a transcription factor (TF) binding site analysis in regions with SA-DNAm changes (450k BeadChip and MethylCap-seq data): 51 motifs were significantly enriched (*P* value < 0.05; Fisher’s Exact test) in senescence-associated DMRs of at least one fibroblast sample or of MSCs. Most of these TF binding motifs were significantly enriched in both hypermethylated and hypomethylated regions (Figure 5A). Significantly overrepresented motifs include binding sites for early growth response protein 1 (EGR1), activating enhancer-binding protein 2 (TFAP2A), protein C-ets-1 (ETS1), neuroblastoma MYC oncogene (MYCN), and aryl hydrocarbon receptor nuclear translocator (ARNT; Figure 5B). Enrichment of these TF binding sites suggests that they are either directly involved in the regulation of SA-DNAm changes or that their binding is influenced by differential methylation and hence relevant for gene expression changes.

Therefore, we analyzed enrichment of TF binding sites in the promoter regions (1 kb upstream) of the differentially expressed genes: 64 motifs were enriched, and most of these were enriched in promoter regions of both up- and downregulated genes (Figure 5C). There is a highly significant overlap of TF motifs enriched in DMRs and differentially expressed genes upon long-term culture (22 motifs marked in bold in Figure 5A,C; *P* value < 10^−4^; Fisher’s Exact test). This enrichment of specific TF binding sites indicates that corresponding factors are relevant for the functional changes during long-term culture.

## Discussion

**Figure 6:**
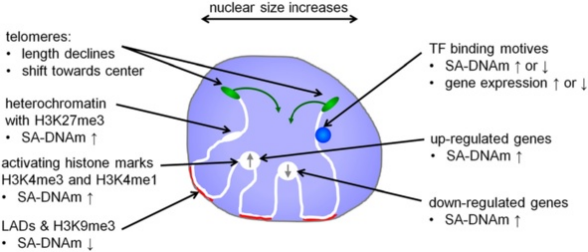
**Scheme of chromosomal changes in replicative senescence.** Nuclear size increases; telomeres shorten and shift towards the nuclear center; DNA hypermethylation is rather observed in regions with the repressive histone mark H3K27me3 and with the activating histone marks H3K4me3 and H3K4me1, whereas hypomethylation is associated with H3K9me3 and LADs; gene expression changes are particularly observed in hypermethylated regions. DNAm changes and differentially expressed genes coincide with binding motifs for specific TFs.

In this study, we demonstrate various facets of the impact replicative senescence has on nuclear organization (Figure 6). This complex picture suggests that different mechanisms are involved in senescence-associated changes, with hypomethylation enriched in inactivated LADs, whereas hypermethylation is reflected by specific changes in gene expression.

Telomere length is well known to decline before cells enter replicative senescence. However, the intranuclear positioning of telomeres, which again reflects a major change in genomic organization, is less clear. It has recently been demonstrated that telomeres are enriched at the nuclear periphery during postmitotic nuclear assembly and become localized at the nuclear center during cell cycle arrest [23]. We also find that telomeres shift away from the nuclear envelope towards the nuclear center at later passages. Transient proximity of telomeres to the nuclear envelope, as well as interaction with A-type lamins, has been suggested to support telomere maintenance, particularly at early passages [28]. In senescent cells, distortion of the ellipsoid-like nuclear shape and lamin A folds protruding into the nucleoplasm have been described [29], which may also contribute to redistribution of telomeres in senescent cells. Such changes in nuclear organization may also entail alterations in the epigenetic make up during cell senescence or *vice versa*.

The DNAm pattern changes during culture expansion in a highly reproducible manner. In fact, an Epigenetic-Senescence-Signature based on DNAm at six specific CpGs even facilitates reliable prediction of passage numbers and cumulative population doublings for quality control of cell preparations [14,30,31]. So far, DNAm changes in replicative senescence were observed in datasets either based on IlluminaBeadChip technology or pyrosequencing of bisulfite-converted DNA. In this regard, it was unexpected that the MethylCap-seq data had relatively little overlap between DNAm changes in the two different fibroblast preparations, even though inter-individual variation can only be estimated because we have only analyzed two samples with this relatively labor-intensive approach. Robust statistical comparison of the MethylCap-seq datasets would require more biological replicates to compensate for the notorious heterogeneity between cell preparations. In this study, we used two available fibroblast preparations that differed slightly in the number of passages at early and late analysis and that this may have contributed to variation between the replicates. However, based on our previous analysis with IlluminaBeadChip technology, which discovered highly consistent DNAm changes that are continuously acquired throughout long-term culture [13,14,19], we would have anticipated a much higher overlap between the two replicates. MethylCap-seq is a robust method for genome-wide DNAm profiling [24,32]. However, reproducibility may be hampered by deviations in DNA fragmentation and efficiency of pull down. The method is biased towards CpG-rich regions [33], and it does not provide DNAm level at a single-nucleotide resolution. Furthermore, results in MethylCap-seq analysis may be influenced by the various parameters in bioinformatics pipelines used for the detection of DMRs. Although SA-DNAm changes are not restricted to individual CpGs, we demonstrated that there is reproducible fluctuation of DNAm in their vicinity, possibly due to a local action of DNA-binding proteins. In addition, age-associated DNAm changes, which are highly reproducible when using the IlluminaBeadChip platform [21,34,35], revealed much lower reproducibility in MethylCap-seq data [36]. Therefore, methods addressing DNAm at a single-site resolution, such as pyrosequencing, MassArray, microarray technology, or whole-genome bisulfite sequencing, may be superior for tracking of specific senescence-associated CpGs. On the other hand, our results based on MethylCap-seq data further validate changes in the DNAm pattern during culture expansion on a genome-wide scale using a different approach, which does not require bisulfite conversion.

We have previously suggested that senescence-associated DNAm changes are related to specific histone modifications by characterizing the promoter regions of genes with SA-CpGs [13]. Further, age-associated hypermethylation is enriched in genes of polycomb group targets, defined by high occupancy of SUZ12, EED, and H3K27me3 in mice [37] and in human [18,34,35,38]. In this study, we specifically analyzed H3K27me3, H3K4me3, H3K4me1, and H3K9me3 profiles at genomic locations of DMRs. There is a moderate enrichment of SA-hypermethylation with H3K27me3 marks in MSCs and a fibroblast sample (donor 2). We have observed an opposite tendency in our previous work [19]. However, the latter analysis was based on H3K27me3 around the promoter of genes close to SA-hypermethylation while our current analysis is based on the regions around the hypermethylated sites.

In all datasets analyzed, we observed significant enrichment of SA-hypermethylation in regions with the activating H3K4me3 and H3K4me1 marks. This is in contrast to a recent study by Fernández and coworkers, who demonstrated that particularly H3K4me1 corresponds to regions that become hypomethylated in MSCs upon aging of the organism [39]. Furthermore, we observed significant enrichment of SA-hypomethylation with H3K9me3 in all datasets analyzed, although these repressive marks were associated with hypermethylated regions upon aging [39]. This supports the notion that DNAm changes in replicative senescence and aging are influenced by independent means [40]. Either way, association of DNAm changes with the histone code confirms that both mechanisms interact and may even be dependent on each other: either the DNAm pattern affects activity of histone modifiers or changes in heterochromatin evoked by the histone code impact on DNAm.

The inner layer of the envelope consists of filamentous proteins, lamin A and C, which are splice variants of the *LMNA* gene, and lamin B1 and lamin B2, encoded by *LMNB1* and *LMNB2*, respectively [41]. Mutations of these genes can affect chromosomal organization [42,43], and such mutations are involved in multiple human diseases, such as cardiac and skeletal myopathies [44] and premature aging [11]. *LMNB1* and *LMNB2* were among the most significantly downregulated genes during culture expansion. In fact, it has been demonstrated that the loss of *LMNB1* is a biomarker for senescence [45], whereas overexpression of LMNB1 increases proliferation and delays onset of senescence in WI-38 cells [46]. Furthermore, it has recently been demonstrated that lamin B1 downregulation in senescence is a key trigger of global and local chromatin changes [47]. The lamin B receptor (*LBR*) was also significantly downregulated. LBR interacts with methyl-CpG-binding protein 2 (MeCP2), the same methylation binding domain we used to capture methylated DNA for MethylCap-seq. This interaction has been suggested to have a role in the localization and/or stabilization of transcriptionally silent heterochromatin adjacent to the nuclear envelope [48]. LADs are implicated in epigenetic regulation due to their relevance for chromosome positioning and influence on chromatin structure [49]. Therefore, remodeling may activate gene expression by moving genes away from the lamina [50]. We demonstrate that loss of DNAm is particularly observed in LADs and that is in agreement with another recent study using whole-genome single-nucleotide bisulfite sequencing in IMR90 cells of early and late passages [17]. It may therefore be speculated that heterochromatin, which is tightly linked to LADs, interferes with accessibility of DNMT1 during cell cycle and hence hypomethylation over subsequent passages [17]. This might mechanistically define epigenetic drift during long-term culture.

In contrast, SA-hypermethylation seems to be associated with differential gene expression of both up- and downregulated genes. These functional changes are reflected by highly specific enrichment of upregulated genes in categories of cellular organization and development, whereas downregulated genes are involved in cell division. Association of DMRs with differential gene expression, even though not necessarily negatively correlated, implies that the SA-hypermethylation may be relevant for these gene expression changes. In fact, several TFs predicted to bind to DMRs and differentially expressed genes upon senescence have been implicated in replicative senescence before: EGR1, also known as zinc finger protein 225, has been shown to play a central role in aging [51,52] and replicative senescence [53]. It has been suggested that deletion of EGR1 leads to a striking phenotype with complete bypass of senescence and apparent immortalization [53]. ETS1, which belongs to the ETS family of downstream targets of the RAS-RAF-MEK signaling pathway, activates the p16INK4a promoter thereby affecting senescence [54]. N-MYC is a proto-oncogene protein that is known to be involved in the regulation of developmental timing in *Caenorhabditis elegans* [55]. Concretely, its binding motif is similar to binding motifs for C-MYC which has also been shown to antagonize senescence and to support reprogramming into the pluripotent state. ARNT forms a complex with ligand-bound aryl hydrocarbon receptor (AhR). Many ligands of the AhR resemble natural and synthetic compounds, some of which are important environmental contaminants [56], indicating that there might be a potential link between environmental influences and senescence. Also, EGR1, MYCN, and ARNT all have a CpG sequence in their core binding motifs. It is conceivable that binding of these TFs is relevant for the regulation of DMR, potentially by interaction with DNA methyltransferases. However, this is not yet conclusive, as hyper- and hypomethylated SA-DNAm changes reveal overlapping enrichment of similar TF binding motifs. Alternatively, SA-DNAm changes play a role to modulate binding of relevant TFs, particularly in hypermethylated regions that coincide with differentially expressed genes.

## Conclusions

In this study, we provide further evidence that epigenetic changes during long-term culture reflect changes in nuclear organization. It remains unclear whether SA-DNAm alterations are due to epigenetic drift or to a tightly regulated process with the possibility that both mechanisms are involved in this process. The finding that SA-hypomethylation is enriched in LADs and H3K9me3 marks without association to specific gene expression changes is compatible with passive and stochastic changes in DNAm level. In contrast, specific SA-hypermethylation is reflected in differential gene expression. Furthermore, the association of SA-DNAm changes with TF binding sites indicates a functionally relevant and controlled process. Notably, both SA-hypermethylation and SA-hypomethylation are reversed when reprogrammed into iPSCs, which may reflect rejuvenation also on the epigenetic level [19]. In this regard, senescence-associated epigenetic modifications seem to be controlled at specific sites in the genome - either actively or passively - and entail the functional changes in the course of replicative senescence. These findings contribute to a better understanding of the molecular process during culture expansion, which hampers standardization of cell preparations in regenerative medicine.

## Methods

### Isolation of primary cells

Human dermal fibroblasts were isolated from patients undergoing surgical interventions after written consent, using guidelines approved by the Ethic Committee on the Use of Human Subjects at the University of Aachen (Permit Number EK163/07) as described in detail before [15]. Cells were culture expanded in DMEM culture medium (PAA Laboratories, Cölbe, Germany; 1 g/L glucose) supplemented with glutamine (PAA), penicillin/streptomycin (PAA), and 10% fetal calf serum (FCS; Biochrom, Berlin, Germany) in a humidified atmosphere at 5% CO_2_. Cells were culture expanded until replicative senescence as determined by ultimate growth arrest. Late passages (as indicated in the text) were within the last three to five passages before entering senescence.

Mesenchymal stromal cells were isolated from the bone marrow of caput femoris upon hip replacement surgery after written consent using guidelines approved by the Ethic Committee on the Use of Human Subjects at the University of Aachen (Permit Number EK128/09) as described before [19]. MSCs were culture expanded in Dulbecco’s modified Eagle’s medium (DMEM) culture medium (PAA) with supplemented with glutamine (PAA), penicillin/streptomycin (PAA), and 10% human platelet lysate (hPL) [57] in a humidified atmosphere at 5% CO_2_. All cell preparations were characterized with regard to immunophenotype and *in vitro* differentiation potential towards osteogenic and adipogenic lineages as described before [15,19].

### Q-FISH analysis of telomeres

Quantitative fluorescent *in situ* hybridization (Q-FISH) was performed on cytospins of three fibroblast preparations of early (P3-5) and corresponding late passages (P 21-40). Staining with a telomere probe labeled with Cy3 (Panagene, Daejeon, Korea) and counterstaining with DAPI was performed as described previously [58,59]. Cell sections were captured in multi-tracking mode (1-μm step size) using a high-resolution Zeiss confocal microscope (LSM710, Zeiss, Jena, Germany). At least 25 nuclei were captured per cell preparation. Definiens XD 2.0 software (Definiens GmbH, Germany) was used for image analysis. Telomere length was calculated by the mean telomere spot intensity with mean background subtraction of the respective nucleus on maximum projection images. To calculate the distance of the detected telomeres in relation to the nucleus, the single *z*-stack image with the largest nuclei area was analyzed. Nuclei were defined in three different zones as recently described [23]. Three zones in the nucleus were defined: border, middle, and center. Nuclear size was normalized (absolute pixel distances) allowing comparison in different nuclei. At least three telomeres had to be detected in one nucleus to be included in the analysis.

### DNAm analysis

DNAm profiles were analyzed by methyl-capture sequencing (MethylCap-seq), which is based on precipitation of methylated DNA by recombinant methyl-CpG binding domain of MeCP2 protein. Fibroblasts from two female donors (both 43 years old) were expanded in culture, and DNA from 10^7^ cells was harvested for subsequent analysis. There was a slight difference in the number of early passages (P3 or P5) due to differences in cell growth, which might be partially attributed to different starting material, and requirement of additional cells for other experiments and long-term culture. The number of corresponding late passages (P30 and P33) was chosen by their growth performance as we wanted to analyze the cells close to senescent state but required residual capability for large-scale expansion. DNA was isolated with the Qiagen DNA Blood Midi-Kit (Qiagen, Hilden, Germany), and quality was assessed with a NanoDrop ND-1000 spectrometer (NanoDrop Technologies, Wilmigton, USA) and gel electrophoresis. DNA was sheared with an S220 focused ultrasonicator (Covaris Inc., Woburn, USA) to a size range of 200 to 400 bp and then incubated with 2 μg of recombinant MBD2-glutathione-S-transferase fusion protein with a histidine tag(H6) [24]. Methylated DNA fragments were then captured on NTA-agarose magnetic beads (Sigma-Aldrich, St. Louis, MO, USA; H9914) and, following washing, eluted by 0.4 M NaCl. Library preparation of methylated DNA fragments and deep sequencing with Illumina technology (IlluminaInc., San Diego, USA) with a read length of 36 bases was performed at EMBL gene core facility (Heidelberg, Germany). Data have been deposited at NCBIs Gene Expression Omnibus (GEO, http://www.ncbi.nlm.nih.gov/geo/; GSE59960). In addition, we used our previously published DNAm profiles of MSCs during long-term culture (GSE37066) [19].

### RNA sequencing

RNA was isolated from 10^6^ cells of three MSC donors (59, 64, and 73 years old) at passage 4 and passage 13 using the miRNeasy Mini Kit (Qiagen, Hilden, Germany). Quality control and measurement of RNA concentration were done with a NanoDrop Spectrophotometer (Thermo Scientific, Wilmington, USA), and the Agilent 2100 Bioanalyzer (Agilent Technologies, Inc., Santa Clara, CA, USA). Multiplexed library preparation of total RNA and deep sequencing with IlluminaHiSeq 2000 technology (Illumina Inc., San Diego, USA) with a read length of 50 bases were performed at EMBL gene core facility (Heidelberg, Germany). RNA-Seq profiles have been deposited at GEO (GSE59966).

### Bioinformatics analysis

Methylcap-seq and RNA-Seq data were subjected to quality control check and preprocessing steps using fastQC (http://www.bioinformatics.babraham.ac.uk/projects/fastqc/) and Flexbar [60]. In all figures, MethylCap, ChIP-Seq, and RNA signals were normalized to obtain reads per kilobase per millions (RPKM) to correct the signal intensities when comparing multiple signals derived from sequencing methods.

For Methylcap-Seq, alignment to the human genome built 37 (hg19) was done with Burrows-Wheeler Transform (BWA) [61]. More than 20 million reads per sample were mapped to the genome. We calculated differentially methylated regions for each donor individually by comparing early passage *versus* corresponding late passage. DMR detection was performed with model-based analysis of ChIP-Seq (MACS; default parameters) [62]. For obtaining hypermethylated regions, we supplied late passage as signal and early passage as control signal. The opposite was performed to obtain hypomethylated regions. We complemented the analysis with H3K4me3, H3K4me1, H3K27me3, and H3K9me3 data (aligned reads) from foreskin fibroblasts from the Epigenomics Roadmap project [25].

The RNA-Seq reads were mapped to the human genome (hg 19) using Bowtie2 [63] and Tophat2 [64]. We used HTSeq [65] with Ensemble 37 (release 71) annotation for quantification of transcripts. Normalization and differential expression analysis were done with DESeq2 [66]. We chose an FDR of 0.01 and a log2 fold change of 2 to detect differentially expressed genes in early or late passage. We used the projection test from the GenometriCorr Package [67] to find associations between DMR signatures and differentially expressed genes.

### Regulatory genomics analysis

Transcription factor enrichment analysis was performed with the Regulatory Genomics Toolbox (http://www.regulatory-genomics.org). Regarding DMRs, we extended or shortened the regions to have a length of 40 bps. For up-/downregulated genes, we used 1-kb regions upstream of the transcription start sites as promoter regions (Ensemble 37, release 71). Next, we performed motif match analysis with a false discovery rate (FDR) of 0.0001 [68]. Motifs were obtained from Uniprobe and Jaspar databases [69,70]. The same procedure was repeated 100 times on random genomic regions with same size of the genomic regions tested. We employed a one-tailed Fisher’s Exact test to measure whether the proportion of binding sites of a motif inside the regions is higher than the proportion of binding sites in random regions. Final *P* values were corrected by the Benjamini-Hochberg method [71].

### Availability of supporting data

Data have been deposited at NCBIs Gene Expression Omnibus (GEO, http://www.ncbi.nlm.nih.gov/geo/; GSE59960 and GSE59966).

## Additional file

Additional file 1: Table S1. Differentially expressed genes.
